# Fractured neck of femur patient care improved by simulated fast-track system

**DOI:** 10.1007/s10195-013-0240-4

**Published:** 2013-04-05

**Authors:** Jonathan D. Kosy, Rachel Blackshaw, Michael Swart, Andrew Fordyce, Robert A. Lofthouse

**Affiliations:** Department of Trauma and Orthopaedic Surgery, Torbay Hospital, Lawes Bridge, Torquay, TQ2 7AA UK

**Keywords:** Fractured neck of femur, Pathway, Optimisation

## Abstract

**Background:**

Fractured neck of femur patients represent a large demand on trauma services, and timely management results in improvements in morbidity and mortality. NICE guidance, advocating surgery on the day of admission or the following day, emphasises this. We set out to investigate whether a simulated fast-track management system could improve neck of femur fracture patient care.

**Materials and methods:**

This prospective study was performed in a district general hospital in South West England, following a change in practise. We studied 429 patients over a 1-year period. Patients were phoned through, by the ambulance crew, to a trauma coordinator who arranged prompt radiological assessment and review. Patients with confirmed fractures were transferred to an optimisation area for orthopaedic and anaesthetic assessment prior to surgery the same day or early the following day. Our primary outcome measures were time to theatre (h) and length of hospital stay (days/h).

**Results:**

Time to theatre reduced from 44.95 (±27.42) to 29.28 (±21.23) h. Length of stay reduced from 10 days (245.92 (±131.02) h) to 9 days (225.30 (±128.75) h). Both of these improvements were statistically significant (*P* < 0.05). Despite operating on virtually all patients, no increase in adverse events was seen, there was no increase in 30-day mortality and there were no perioperative deaths.

**Conclusions:**

This coordinated management pathway improves the efficiency of the service and reduces inpatient length of stay. Increased productivity may lead to financial savings and improve our ability to meet guidelines.

## Introduction

Each year there are between 700,000 and 750,000 hip fractures in the UK, with an associated estimated cost of medical and social care of £2 billion [1]. These figures will further increase with an ageing population. Fractured neck of femur patients represent a large burden on trauma service and are associated with significant mortality: approximately 10 % at 1 month and 33 % at 1 year postfracture [1]. Despite this, these patients are often given low priority, resulting in increasing demands on trauma care and the planning of subspecialty work [2]. Pain control with opiates is difficult and results in unwanted sedative effects. Starvation, cancellation and delay leads to significant morbidity for elderly, frail patients and creates considerable demands in terms of difficult nursing and prolonged rehabilitation [2]. It has even been argued that the elderly patient with a fracture of the hip seems to be somewhat forgotten [2].

Recently published meta-analysis data show clear improvements in the mortality at 1 year and a reduction in complications associated with early surgery [3]. This has been highlighted in guidance from the National Institute for Health and Clinical Excellence (NICE), published in June 2011, advising that surgery should be undertaken on the day of admission or the following day [1].

In our medium-sized district general hospital, serving a population of 300,000 patients (with a high number of elderly residents), prior to this work there was a time delay from admission to theatre of 45 h. Significant numbers of the trauma beds were occupied by these patients, with a total length of stay of 10 days. Patients stated that they were waiting too long and in pain for an operation. It was recognised that the entire admission and management process of this patient group needed to be addressed to improve this.

## Materials and methods

We performed a process simulation, involving representatives of all of the healthcare professionals involved (orthopaedic surgeons, anaesthetists, ward nurses, emergency nurses, intensivists, orthogeriatricians, theatre staff), as well as patients. The simulation showed that the priority, as the diagnosis was evident to the ambulance crew, was to get radiographic confirmation and provide effective ongoing analgesia. This led to the creation of the trauma coordinator role (Table 1). It was noted that there were significant delays in the emergency department and radiology. During this time, the patient’s pain was poorly controlled.

**Table 1:** Defining the trauma coordinator role

Trauma coordinator role
Senior nurse with ward-management experience
Competent to cannulate and take blood
Completion of ionising radiation (medical exposure) regulations training so that X-ray requests can be completed
Training in the provision of local anaesthetic blocks
Limited prescribing rights (to include analgesics)

**Fig. 1:**
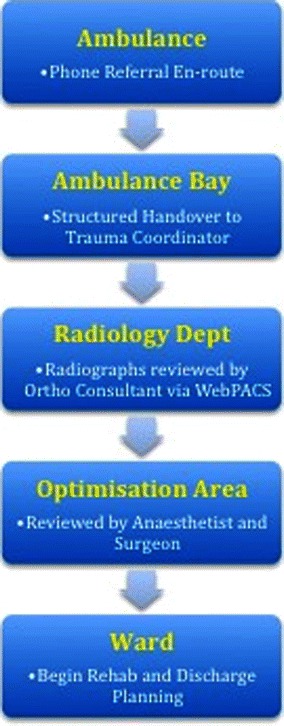
Redesigned pathway

Following the simulation, the project team mapped an ideal process (Fig. 1).

Most of the necessary tasks, which were previously performed in the emergency department, could be done more efficiently by other members of the team (and thus without the significant delays found in this department). Therefore, this part of the admission process was eliminated (unless there was a specific indication of a need for the skills of an emergency specialist). The resultant pathway is shown in Table 2.

**Table 2:** Summary of the management pathway

Pathway
*Prior to hospital*
Paramedic team:
Contacts trauma coordinator en route by phone
Obtains intravenous access
Administers appropriate analgesia
Performs ECG
*On arrival at hospital*
Trauma coordinator arrives at ambulance bay and receives standardised handover:
Identifies emergent medical issues
Gathers key social information essential for the discharge process
*Prior to X-ray*
Trauma coordinator:
Completes X-ray request form
Scores pain level and gives analgesia
Starts pathway paperwork, including:
MRSA risk
Diarrhoea and vomiting assessment
Refers to emergency department team if there are any medical concerns
*After X-ray*
Fracture confirmed by on-call orthopaedic consultant using picture archiving and communication system (PACS)
Patient moved to optimisation area
*In optimisation area (bay in recovery suite of theatre complex)*
Local anaesthetic block (fascia iliaca block) performed by trauma co-ordinator
Review performed by operating surgeon and anaesthetist
Review done by intensivist if required
Prepared for surgery on trauma list or elective list (where space available)
If no surgical time available, transferred to ward
*Following surgery*
Transferred to ward

**Fig. 2:**
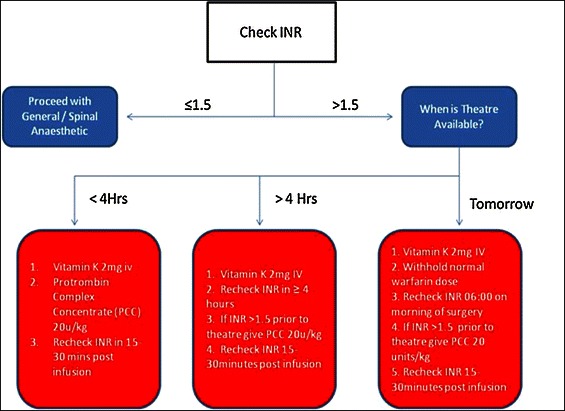
Anticoagulation pathway

Two particular areas of concern were raised and specific plans were made. Following discussion with the haematology department, a protocol for the management of anticoagulated patients was created (Fig. 2). This uses a prothrombin complex concentrate to reverse the effects of warfarin and allow immediate, safe surgery (when theatre space is available). Secondly, situations where the patient is unable to give consent were discussed. It was decided that, in this instance, when efforts to contact the immediate family failed, surgery would be delayed.

This new pathway has been used for all patients admitted to our hospital with a fractured neck of femur from 22nd November 2010. The trauma coordinators collected prospective data on all of the timings involved, and this was inputted daily into a database.

In the 12 months following the change, we treated 429 consecutive patients using this pathway [mean age 80.2 (±12.7) years]. No patients were excluded.

This data was compared to retrospective data for the 6 months prior to the change in practise—211 patients [mean age 81.3 (±12.1) years].

Results were compared using an unpaired, one-tailed *t* test.

In our post-change prospective series, only 4 patients were rejected for surgical treatment, as they were moribund. This number is thought to be considerably less than previously experienced (although we do not have accurate data about the number of patients who were refused surgery before this change).

Follow-up of each of the patients in the study was performed by the trauma coordinators. Thirty days postsurgery, the patients were contacted (or contact was made with the institution in which they were resident) to obtain information on mortality. This process of contacting the patient or institution was repeated at the 1-year stage.

## Results

The mean time to theatre initially reduced from 44.95 (±27.42) to 29.25 (±21.23) h. This was statistically significant (*P* < 0.001).

The mean length of stay from fracture to discharge from our hospital has decreased from 10 days [245.92 (±131.02) h] to 9 days [225.30 (±128.75) h]. This was statistically significant (*P* < 0.05).

The median changes in both our time to theatre and length of stay and are summarised, along with the other results, in Table 3. The improvements in the median values are less affected than the mean values by the skew of a small number of patients who suffered significant delays in being operated on or being discharged. We would suggest that this implies larger improvements in our figures than the mean values show.

**Table 3:** Summary of results

	Statistic	Time to theatre (h)	Length of stay
Pre-change	Mean (± SD)	44.95 (±27.42)	245.92 (±131.02) h
	Median (range)	42 (2–159)	10 (2–39) days
Post-change	Mean (± SD)	29.28 (±21.23)	225.30 (±128.75) h
	Median (range)	22 (2–78)	7 (2–20) days
	*t* test	*P* < 0.001	*P* = 0.0294

During this time we have had no intraoperative deaths. There has been no change in our 30-day mortality rate (6.4 %).

The improvements produced by this change are displayed in Table 4.

**Table 4:** Gap analysis showing changes

Measure	Where we were	Where we are
Median time to theatre	42 h	22 h
% Treated on day of injury	3 %	33 %
% Treated in 24 h of admission	29 %	71 %
% Treated in 48 h	66 %	90 %
Median length of stay	10 days	7 days
Cost saving estimate		£326,000 per year

## Discussion

Fractured neck of femur patients have traditionally been given low priority, with avoidable delays to surgery occurring in up to 55 % of patients [2]. It has been widely believed that these patients need admission for “optimisation” prior to surgery, with days spent performing further investigations or initiating additional treatments by continually changing surgical and anaesthetic teams. The effect of these delays has been debated in terms of changes to the morbidity and mortality of these patients. Weller et al. reported a series of 57,315 patients from the Canadian Audit database who showed increased in-hospital mortality with a 24 h delay (odds ratio 1.13). The in-hospital mortality further increased when the delay exceeded 48 h (odds ratio 1.6) [4]. This increase in mortality was reflected in other studies [5–8].

Although patients with pre-existing medical co-morbidities have been shown to have a worse prognosis following surgery [9], taking time to correct abnormalities does not seem to be advantageous. In a study by Holt et al. [10] of 4,284 registry patients from Scotland, patients with major co-morbidities were—unsurprisingly—more likely to be postponed. However, they were unable to demonstrate that postponement led to any improvement in survival (with a subgroup doing even worse) [10].

The incidence of complications of bed rest and the development of medical problems postoperatively has also been investigated. Parker et al. showed statistically significant increases in pressure sores in delayed patients and a trend towards increases in the rates of pulmonary emboli and pneumonia [11]. Lefaivre et al. showed that this increase in major complications is greater than twofold if the delay exceeds 48 h [12].

In the largest meta-analysis in the literature (including a total of 13,478 patients), Simonovic and colleagues reported that early surgery decreases mortality at 1 year by 45 % and reduces in the incidence of postoperative pneumonia (relative risk 0.59) and that of pressure sores (relative risk 0.48) [3].

Our data show no increase in our intraoperative, in-hospital, or 30-day mortality rates. This is in keeping with previous studies.

Rehabilitating neck of femur patients and reducing inpatient stay are also of importance. Previous studies have shown early surgery results in better mobilisation postoperatively, and this should translate into earlier discharge [13, 14]. Siu et al. showed that bed rest led to a deleterious effect on muscle quality, and showed a one-point difference in the motor scale of the functional independence measure [15]. They concluded that this can be the difference between the patient going home independently and needing assistance from a carer to mobilise (thus requiring ongoing institutional care) [15].

Our reduction in the length of hospital stay (median 10–7 days) indicates that the decrease in hospitalisation time is far greater than just the time saved preoperatively, as our median time-to-theatre decreased by only 20 h (median 42–22 h). Patients are visibly more able to sit out of bed on postoperative day 1, and are more able to partake in physiotherapist-led rehabilitation at this stage.

In setting up this project, we encountered concerns about it being too dangerous to anaesthetise patients without additional investigations, and about having to delay other operations to create additional theatre time for these neck of femur patients. Both of these problems have been shown to be unfounded. Techniques allow for a safe and effective anaesthetic to be performed for virtually all of our patients, and problems such as cardiac function and fasting status can be safely factored into the tailoring of the anaesthetic. We have been able to fit over 50 % of cases into existing lists without the need to cancel patients. However, we have found that for practicality purposes, the patient must arrive at the emergency department before 14:00 to allow for investigations and arrangements to be made. Pivotal to overcoming some of the scepticism has been involvement of the whole team in the modelling and simulation processes and in reporting ongoing improvements as they occur.

Our experience is that the role of the trauma coordinator is essential. Other vital considerations were the positioning of the optimisation area and the management of anticoagulated patients. The optimisation area (in our hospital, this is a bay in the theatre recovery suite) needs to be within the theatre complex. This allows prompt review of the patient by the surgical and anaesthetic teams around existing theatre cases, and prevents delays associated with admitting the patient to the ward and arranging transfer to theatre. Finally, agreeing on the use of a prothrombin complex concentrate to reverse anticoagulated patients and allow immediate surgery has removed the previously time-consuming process of reversal and rechecking of blood samples.

Although we have not specifically measured these effects, there appear to have been many additional benefits felt by other members of the multidisciplinary team. The ward nurses report fewer preoperative neck of femur fracture patients who require transfer onto bedpans and around the bed space in pain. The physiotherapists are able to mobilise patients much more successfully on day 1 postoperatively, and have noted that this results in the patients progressing to a safe, independent level of mobility more rapidly. The surgical ward round now has the pleasure of reviewing postoperative patients who are planning on getting home rather than preoperative patients who are getting increasingly unwell and frustrated by delays. Finally, the patients and their families seem delighted by the level of service and prompt care they are receiving and are very complimentary in their comments.

Other improvements that we have noticed within the orthopaedic department include improved trauma flow. As the neck of femur fractures are managed more efficiently, fewer cases are waiting for surgery each morning. In addition, our ability to plan complex subspecialty cases has improved, as we are able to provide more time on the trauma lists with subspecialists (this time was previously occupied by fractured neck of femur procedures). Prompt starts to trauma lists are possible, as the patient has often been fully worked-up in the optimisation area on the previous day and can be operated on first.

Financial benefits are difficult to prove. However, the decreased length of stay means that we are able to increase the productivity of each hospital bed. This should equate to savings of hundreds of thousands of pounds. The savings in terms of overall healthcare expenditure, including ongoing costs of care in community hospitals and residential/nursing homes, may be even more significant.

Therefore, without specifically focusing on reducing either time to theatre or length of stay, we have redesigned our management of fractured neck of femur patients using a patient-focussed approach. This had been achieved without the need for additional personnel, theatre space or resources. We are now in a much stronger position to meet the recent NICE guidance advocating surgery the same day (or the following day).

## References

[1] National Institute for Health and Clinical Excellence (2012) Hip fracture—the management of hip fracture in adults. Available at: http://guidance.nice.org.uk/CG124. Accessed 28 Aug 2012

[2] Von Meibon N, Gilson N, Dhapre A, Davis B (2007). Operative delay for fracture of the hip: a two-centre prospective study. J Bone Joint Surg [Br].

[3] Simunovic N, Devereaux PJ, Sprague S (2010). Effect of early surgery after hip fracture on mortality and complications: a systematic review and meta-analysis. CMAJ.

[4] Weller I, Wai EK, Jaglal S (2005). The effect of hospital type and surgical delay on mortality after surgery for hip fracture. J Bone Joint Surg [Br].

[5] Hamlet WP, Libermann JR, Freedman EL (1997). Influence of health status and the timing of surgery on mortality in hip fracture patients. Am J Orthop.

[6] Villar RN, Allen SM, Barnes SJ (1986). Hip fractures in healthy patients: operative delay versus prognosis. Br Med J (Clin Res Ed).

[7] Bredahl C, Nyholm B, Hindsholm FB (1992). Mortality after hip fracture: results of operation within 12 h of admission. Injury.

[8] Zuckerman JD, Skovron ML, Koval KJ (1995). Postoperative complications and mortality associated with operative delay in older patients who have a fracture of the hip. J Bone Joint Surg Am.

[9] Moran CG, Wenn RT, Sikand M (2005). Early mortality after hip fracture: is delay before surgery important?. J Bone Joint Surg Am.

[10] Holt G, Smith R, Duncan K (2010). Does delay to theatre for medical reasons affect the peri-operative mortality in patients with a fracture of the hip?. J Bone Joint Surg [Br].

[11] Parker MJ, Pryor GA (1992). The timing of surgery for proximal femoral fractures. J Bone Joint Surg [Br].

[12] Lefaivre KA, Macadam SA, Davidson DJ (2009). Length of stay, mortality, morbidity and delay to surgery in hip fractures. J Bone Joint Surg [Br].

[13] Siu AL, Penrod JD, Boockvar KS (2006). Early ambulation after hip fracture: effects on function and mortality. Arch Intern Med.

[14] Gill TM, Allore HG, Holford TR (2004). Hospitalisation restricted activity, and the development of disability among older persons. JAMA.

[15] Turner-Stokes L, Nyein K, Turner-Stokes T (1999). The UK FIM + FAM: development and evaluation. Clin Rehabil.

